# P315: Sexual risk behaviors among minority men who have sex with men with and without HIV

**DOI:** 10.1186/2047-2994-2-S1-P315

**Published:** 2013-06-20

**Authors:** K Gordon, J Edelman, A Justice, S Crystal, D Rimland, M Duggal, D Fiellin, K Bryant

**Affiliations:** 1 VACHS, West Haven, CT, USA; 2 Rutgers, New Brunswick, NJ, USA; 3 Emory, Atlanta, USA; 4 Yale, New Haven, CT, USA; 5 NIAAA-NIH, Bethesda, MD, USA

## Introduction

Minority men who have sex with men (MSM) have the highest incidence of human immunodeficiency virus (HIV) infection. They also have high alcohol use, which is associated with sexual risk behaviors and detectable HIV-RNA levels; both of which increase the risk of HIV transmission.

## Objectives

We hypothesized that minority MSM would be associated with sexual risk behaviors, and, among HIV-infected men, detectable HIVRNA.

## Methods

We used data from the Veterans Aging Cohort Study, an observational cohort study of HIV+ and matched uninfected patients. MSM status and race/ethnicity were based on self-report. We performed descriptive and multivariable logistic regression analyses to determine the association between MSM/race (minority MSM, white MSM, minority non-MSM and white non-MSM) and sexual risk behaviors (multiple sex partners, sex under the influence of drugs/alcohol and non-condom use). All analyses were adjusted for demographics and medical characteristics.

## Results

Among the 6,175 male participants, 51% were HIV+, 27% were MSM (18% minorities), and 36% were hazardous drinkers or diagnosed with alcohol abuse/dependence. Minority MSM were more likely to have multiple sex partners, and be under the influence of alcohol/drugs during sex, compared to all other groups. They were also more likely to have hazardous drinking and alcohol abuse/dependence. Moreover, HIV+ minority MSM were more likely to have higher median HIV RNA. In adjusted logistic regressions, minority MSM, compared to white non-MSM, were more likely to have multiple sex partners (AOR=6.3[4.4, 9.0]), be under the influence of alcohol/drugs during sex (AOR=2.1[1.4, 3.0]) and report non-condom use (AOR=1.1 [0.8, 1.4]). Among HIV+, minority MSM were more likely to have a detectable viral load (HIVRNA≥500 copies/mL) (AOR=1.5 [1.0, 2.3]).

## Conclusion

These analyses suggest that a minority MSM with alcohol abuse/dependence would be 11.5[6.4, 20.4] more likely to have multiple sexual partners and 19.6[10.4, 37.0] more likely to have sex under the influence, making minority MSM an extreme risk group. Our data support the need for tailored prevention efforts targeting these behaviors among minority MSM.

## Disclosure of interest

None declared

